# Strabismus surgery in Angelman syndrome: More than ocular alignment

**DOI:** 10.1371/journal.pone.0242366

**Published:** 2020-11-13

**Authors:** Paola Michieletto, Stefano Pensiero, Laura Diplotti, Luca Ronfani, Manuela Giangreco, Alberto Danieli, Paolo Bonanni

**Affiliations:** 1 Ophthalmology Service, Scientific Institute IRCCS Eugenio Medea, Conegliano-Pieve di Soligo (TV), Italy; 2 Department of Ophthalmology, Institute for Maternal and Child Health—IRCCS Burlo Garofolo, Trieste, Italy; 3 Clinical Epidemiology and Public Health Research Unit, Institute for Maternal and Child Health—IRCCS Burlo Garofolo, Trieste, Italy; 4 Epilepsy and Clinical Neurophysiology Unit, Scientific Institute IRCCS Eugenio Medea, Conegliano-Pieve di Soligo (TV), Italy; Faculty of Medicine, Cairo University, EGYPT

## Abstract

**Purpose:**

To report and evaluate strabismus surgery in children with Angelman syndrome, in order to optimize and standardize surgical approach. Other purposes are to understand the possible relation between ocular findings and motor ability, and between improvement in ocular alignment and changes in motor skills in this population.

**Design:**

Observational cross-sectional study.

**Methods:**

Medical records of pediatric patients with Angelman syndrome, who underwent strabismus surgery, were investigated. Collected data included: genotype, gender, age at the time of surgery, refractive error, pre-operative strabismus, surgical procedure, surgical outcome, gross and fine motor development assessment pre- and post-operatively.

**Results:**

Seventeen subjects, aged 3–15 years, were investigated. Fourteen patients were exotropic, three esotropic. Most patients presented astigmatism. Considering the exaggerated response to standard amounts of surgery and the risk of consecutive strabismus on long term follow-up reported by previous studies in children with developmental delay, a reduction of the amount of strabismus surgery was applied. Post-operatively, all patients presented with a significative reduction of the baseline deviation angle, with all esotropic patients and 7 exotropic patients (59%) achieving orthotropia. The surgical outcomes were variable according to the type and the amount of baseline strabismus, but no case presented with exaggerated surgical response. At baseline, patients showed important delays in all motor abilities, and, post-operatively, presented a significant improvement in walking and fine motor tasks. Pre- and post-operative motor abilities were negatively correlated to astigmatism, anisometropia, and amount of deviation.

**Conclusions:**

According to our data, the standard nomograms for strabismus surgery may be successfully applied in subjects with Angelman syndrome and exotropia. Our data suggest that the reduction of the deviation angle improves motor skills in strabismic pediatric patients with Angelman syndrome.

## Introduction

Angelman syndrome (AS) is a rare genetic neuro-behavioural disorder, affecting between 1/15.000 and 1/20.000 people worldwide, with variable expressivity and penetrance [1]. It is caused by various inactivating mutations of the *UBE3A* gene on the 15q11.2–13.3 chromosomal region [2–4], including: *de novo* interstitial deletions of maternally-inherited chromosome 15 (class I, 70–75%, often resulting in more severe phenotypes [5, 6]), paternal uniparental disomy of chromosome 15 (class II, 2–3%), paternal imprinting disorders in chromosome 15 (class III, 3–5%), point mutations in the maternally-inherited *UBE3A* gene (class IV, 5–10%), and currently unknown genetic mechanisms (class V, 10–15%) [1, 7].

AS is usually characterized by delayed development, intellectual disability, speech impairment, ataxia, epilepsy and/or abnormal electroencephalography, microcephaly, scoliosis, and a typical behavioural profile, i.e. hyperactivity, an apparently happy demeanour with frequent smiling and laughter [8, 9]. As clinical features show wide inter-individual variability, often overlapping with other neuro-behavioural conditions, AS may be difficult to diagnose, especially in the first years of life [1]. The diagnosis of AS is based on clinical, behavioural and developmental features [10] and is confirmed by laboratory genetic tests [11].

Despite there is abundant literature documenting the clinical and neuro-behavioural profiles of AS, a relative paucity of information regarding ocular manifestations of AS and their possible treatment is currently available [12–16]. Ophthalmic findings in AS include ametropia (typically astigmatism), strabismus (usually exotropia) and less frequently nystagmus, amblyopia, iris and choroidal hypopigmentation, optic nerve atrophy or optic disk pallor, retino-choroidal atrophy, ptosis and keratoconus [16].

Considering the high prevalence of ocular motility and refractive disorders found in their study (75% and 97%, respectively), Michieletto and colleagues [16] suggested that early diagnosis and prompt treatment of ocular alterations should be part of the rehabilitative program of AS individuals, as it could have positive effects on cognitive aspects. Drover et al. [17] already suggested that surgical correction of infantile strabismus in children may positively influence visual and motor development, but little has been published about children with developmental delays [18–25]; and even lesser with regards to AS [26].

Surgical planning can be difficult in children with AS, since clinical evaluation and precise strabismus assessment are often challenging [26], standard strabismus surgery presents scarce and unpredictable outcomes in delayed children [27], yet, in the scientific literature there is no consensus on the most suitable surgical dosage adjustment [18–24]. Anesthesia planning can also be demanding in AS subjects, as complex airway management due to craniofacial abnormalities, atypical responses to benzodiazepines and malignant bradydysrhythmias during anesthesia have been reported [28]. Finally, even the evaluation of surgical outcomes can be critical in AS subjects, that usually show motor function impairment, cognitive and communicative disability and inattention. In this regard, overall, in the literature, there are only limited data about the developmental profile of AS subjects and its change over time [29–31], and no data at all comparing mental and motor development with ocular motility, refractive state, and their correction.

The aim of this study is to report the effect of strabismus surgery in children with AS and the results of motor development assessment performed before and after surgery, in order to evaluate the surgical outcomes, the possible relation between ocular findings and motor ability, and between improvement in ocular alignment and changes in gross and fine motor skills.

## Material and methods

Medical records of 17 pediatric patients with AS, who underwent strabismus surgery between 2014 and 2018 were investigated. All patients were referred to the Scientific Institute IRCCS Eugenio Medea, Conegliano-Pieve di Soligo (TV, Italy) for neurobehavioral rehabilitation and underwent surgical correction of strabismus at the Institute for Maternal and Child Health of Trieste—IRCCS Burlo Garofolo (TS, Italy). The research was approved by the Institutional Review Board of the IRCCS Burlo Garofolo and adheres to the tenets of the Declaration of Helsinki.

All naïve patients underwent strabismus surgery under general anesthesia with endotracheal intubation and assisted ventilation. All patients underwent a complete ophthalmological and orthoptic examination both before and after operation. The simultaneous prism and cover test was performed to assess and quantify the baseline and final deviation angle. Before surgery, all clinically significant refractive errors were treated, even though often AS children don’t wear spectacles permanently, as prescribed.

When operating healthy children without high refractive errors, we usually calculate the amount of surgery according to the following rule: for esotropic patients 1 mm MR recession for every 3 PD of deviation angle (up to a maximum of 6 mm recession), eventually combined with 1 mm ML resection for every 3 PD (up to 5 mm resection); for exotropic patients 1 mm LR recession for every 2 PD of deviation angle (up to a maximum of 8 mm recession), eventually combined with 1 mm MR resection for every 3 PD (up to 5 mm resection). Where possible, we performed symmetrical bilateral surgery.

This approach results from the adaptation of the nomograms proposed in the scientific literature to our personal experience and is superimposable to the guidelines by Taylor et al. [32]. As precise strabismus assessment and surgical planning is often challenging in AS patients, especially in subjects with greater deviations, and considering the exaggerated response to standard amounts of surgery [21] and the risk of consecutive strabismus on long term follow-up [22–25] reported by previous studies, we applied a small reduction of the amount of surgery to correct esotropia and a greater reduction to correct exotropia (i.e. we performed only 8 mm bilateral LR recession for 45 PD exotropic correction).

The Gross Motor Function Measure (GMFM [33]) and the Bayley Scales of Infant and Toddler Development—III Edition (BSID-III [34]) were used to assess gross and fine motor development.

GMFM is a standardized observational instrument designed to measure change over time in the gross motor function of children with cerebral palsy aged 5 months to 16 years [33]. BSID-III is a comprehensive quantitative assessment tool, based on a series of standardized tasks, for determining cognitive, language, and motor development in infants and toddlers aged 1–42 months [34]. In previous studies, both GMFM and BSID-III test have also been administered in subjects with developmental disabilities of all ages, beyond clinical indications [35].

Clinical data collected included: class of genetic mechanisms causing AS (I-IV), gender, age at the time of surgery, cycloplegic refraction, pre-operative strabismus, surgical procedure, surgical outcome 1 year post-operatively, gross and fine motor development assessment pre-operatively and 3 months after surgery. The following variables were taken into consideration to evaluate motor abilities: GMFM items “Lying and Rolling” (LyR), “Sitting” (Si), “Crawling and Kneeling” (CK), “Standing” (St), “Walking, running and jumping” (WRJ), “Total score” (G-TS) and BSID-III “Total score” (B-TS) index.

As children with neurological impairment are usually unable to complete conventional visual acuity tests and sensory fusion tests, it was not possible to collect these data in our cohort: best corrected visual acuity (BCVA) was only measured bilaterally in 3 patients and we were unable to assess binocular single vision at all.

Descriptive statistics were used to report the results. Categorical variables are presented as numbers and percentages, continuous variables as median and interquartile range (IQR). Fisher's exact test and McNemar test were used to evaluate associations between categorical variables and matched categorical variables, respectively. Wilcoxon-Mann-Whitney test and Kruskal-Wallis test were applied to assess the difference in distribution of a continuous variable across the groups of a categorical variable (two groups or more than two groups, respectively). Spearman's test was used to calculate the correlation between two continuous variables and Wilcoxon signed-rank test to test whether two matched samples derived from populations with the same distribution. Statistical significance was defined as P-value (*P*) < 0.05.

All statistical analysis was conducted using SAS software, Version 9.4 (SAS Institute Inc., Cary, NC, USA).

## Results

The main results are provided in Tables 1 and 2. Overall, 17 patients, 7 (41.2%) males and 10 (58.8%) females, aged between 3 and 15 years (median: 9 years; IQR: 8–12.5 years), were enrolled in this study. According to genotype, 12 patients (70.6%) were affected by Class I AS, 1 (5.9%) by Class II, 1 (5.9%) by Class III, and 3 (17.6%) by Class IV. No association was found between gender and AS class (all *p* > 0.05).

**Table 1:** Ophthalmologic characteristics of the enrolled population.

ID	Class^a^	Gen	Age (y)	RE refractive error (D)	LE refractive error (D)	B BCVA	Pre-op dev (PD) ^b^		Type and amount of surgery (mm)	Post-op dev (PD) ^b^	
1	I	F	13	+2.00	+2.00	/	+18	RE	RE: MR rec 5	ort	
2	I	F	9	+1.00 +2.00/90	+0.50 +1.25/95	/	-40	int	B: LR rec 8	ort	
3	I	F	12	+2.50	+2.00 +1.00/100	/	-45	alt	B: LR rec 8	-25	alt
4	I	M	8	+1.50 +2.50/90	+1.50 +3.50/75	/	-45	alt	B: LR rec 8	-20	alt
5	I	F	9	-1.25 +2.50/110	+0.25 +1.25/60	/	-35	LE	B: LR rec 7	-12	LE
6	I	M	13	+3.00 +0.50/80	+2.00 +1.50/80	/	-35	LE	B: LR rec 7	-25	LE
7	I	M	3	+1.50 +1.00/100	+1.50 +1.25/80	/	-30	int	B: LR rec 7	ort	
8	I	F	12	+2.50 +4.00/100	+2.75 +3.75/70	/	-30	RE	B: LR rec 7	-12	RE
9	I	F	9	+1.50 +2.50/90	-3.00 +3.50/180	/	-45	RE	B: LR rec 8	-16	RE
10	I	M	14	+1.25 +2.75/105	+1.25 +2.25/90	/	-30	LE, ny	LE: LR rec 6, MR res 4	ort	
11	I	F	8	-3.25 +3.25/120	-2.00 +2.25/105	/	-60	RE	B: LR rec 8, MR res 4	-14	RE
12	I	F	5	+0.25 +2.50/105	+1.00 +2.50/95	/	-50	int, V	B: LR rec 8, B: IO rec	ort	
13	II	F	9	+1.50	+1.25	20/25	-30	int	B: LR rec 7	ort	
14	III	M	8	+1,00 +0.75/100	+0.50 +1.00/105	/	-25	int	B: LR rec 6	ort	
15	IV	F	9	-0.50	-0.25	20/40	-20	int	B: LR rec 5	ort	
16	IV	M	4	+4.50 +1.25/90	+3.50 +2.25/100	/	+12	V	LE: MR rec 4, B: IO rec	ort	
17	IV	M	15	-1.50 +1.50/80	-1.50/170	20/50	+30		B: MR rec 4.5	ort	

ID = patients’ identification number; Class = genotype (genetic mechanisms causing Angelman syndrome); Gen = gender; F = female; M = male; y = years; RE = right eye; LE = left eye; B = bilateral; Pre-op = pre-operative; Post-op = post-operative (1 year after operation); dev = deviation; D = dioptres; PD = prism dioptres; BCVA = best corrected visual acuity; int = intermittent; V = V pattern; ny = nystagmus; MR = medial rectus muscle; LR = lateral rectus muscle; IO = inferior oblique muscle; rec = recession; res = resection, ort = orthotropia (deviation < 8 PD); alt = alternating; / = not available

^**a**^ Class: I–de novo interstitial deletions on maternally-inherited chromosome 15q11.2–13.3; II–paternal uniparental disomy of chromosome 15; III–paternal imprinting disorders in chromosome 15; IV–point mutations in the maternally-inherited *UBE3A* gene.

^**b**^ Positive numbers refer to esodeviations; negative numbers to exodeviations.

**Table 2:** Motor development assessment in the enrolled population.

ID	Class^a^	Gen	Age (y)	GMFMS												BSID-III	
				LyR (%)		Si (%)		CK (%)		St (%)		WRJ (%)		TS (%)		TS (%)	
				pre	post	pre	post	pre	post	pre	post	pre	post	pre	post	pre	post
1	I	F	13	94	88	97	85	93	79	72	59	35	36	78	69	21	/
2	I	F	9	88	/	70	/	7	/	15	/	6	/	37	/	26	/
3	I	F	12	100	100	95	98	71	79	74	74	56	60	79	82	40	40
4	I	M	8	76	76	72	85	7	7	67	72	31	40	51	56	26	26
5	I	F	9	88	88	72	73	14	12	13	13	13	14	40	40	21	21
6	I	M	13	100	/	100	/	86	/	74	/	53	/	83	/	26	30
7	I	M	3	98	/	60	/	7	/	10	/	8	/	37	/	26	29
8	I	F	12	88	94	98	98	71	79	67	72	36	53	72	79	28	30
9	I	F	9	75	76	20	20	5	5	0	0	0	0	20	20	21	23
10	I	M	14	100	/	100	/	81	/	79	/	60	/	84	/	38	/
11	I	F	8	100	100	73	73	7	7	54	59	50	50	57	58	28	30
12	I	F	5	/	/	/	/	/	/	/	/	/	/	/	/	/	/
13	II	F	9	/	/	/	/	/	/	/	/	/	/	/	/	/	/
14	III	M	8	100	/	100	/	100	/	77	/	42	/	84	/	40	/
15	IV	F	9	100	100	100	100	86	95	85	85	71	75	88	91	47	47
16	IV	M	4	100	100	85	100	74	88	69	72	28	51	71	82	30	38
17	IV	M	15	/	/	/	/	/	/	/	/	/	/	/	/	/	/

ID = patients’ identification number; Class = genotype (genetic mechanisms causing Angelman syndrome); Gen = gender; F = female; M = male; y = years; GMFMS = Gross Motor Function Measure Scale; BSID-III = Bayley scales of infant and toddler development, 3rd edition; LyR = Lying and Rolling; Si = Sitting; CK = Crawling and Kneeling; St = Standing; WRJ = Walking, running and jumping; TS = Total score; pre = pre-operative; post = post-operative (3 months after operation); / = not available.

^**a**^ Class: I–de novo interstitial deletions on maternally-inherited chromosome 15q11.2–13.3; II–paternal uniparental disomy of chromosome 15; III–paternal imprinting disorders in chromosome 15; IV–point mutations in the maternally-inherited *UBE3A* gene.

The ophthalmologic characteristics of all patients are provided in Table 1.

Overall, 15 (88.2%) patients presented a refractive error greater than 1 Diopters (D): 10 (71.4%) had compound hyperopic astigmatism, 3 (21.4%) mixed astigmatism, 1 (7.2%) myopic astigmatism and 1 (6.7%) mild hyperopia. No case of keratoconus was observed.

Visual acuity measurement was only available for 3 patients (none of them with class I AS).

Refractive errors and anisometropia were neither significantly different between genetic classes (all *p* > 0.05) nor correlated with age (all *p* > 0.05).

At baseline, esotropia was present in 3 (17.6%) patients, including a case of V-pattern strabismus, with an average deviation of 18 (IQR: 12–30) prism dioptres (PD). All esotropic patients underwent uni- or bilateral medial rectus muscle (MR) recession, which was associated with bilateral inferior oblique muscle (IO) recession in the patient with V-pattern strabismus. Post-operatively all esotropic patients achieved orthotropia (deviation < 8 PD).

Exotropia was present in 14 (82.4%) patients, including 2 cases of large angle alternating strabismus, 5 cases of intermittent strabismus and 1 case of V-pattern intermittent strabismus, with an average deviation of 35 (IQR: 30–45) PD. All patients underwent uni- or bilateral lateral rectus muscle (LR) recession, which was combined with IO recession in the patient with V-pattern strabismus. LR recession was associated with MR resection unilaterally in Subject 10, who had 30 PD monocular exotropia and nystagmus; bilaterally in Subject 11, who had 60 PD monocular exotropia (the greater deviation angle in our cohort). Postoperatively 7 (50.0%) patients achieved orthotropia, 5 (35.7%) patients presented 12–25 PD residual monocular exotropia and 2 (14.3%) patients presented 20–25 PD residual alternating exotropia. Subject 6, who had 35 PD monocular exotropia, presented 12 PD residual monocular exotropia 3 months post-operatively, which increased to 25 PD at 6 months follow-up, and then stabilized up to 12 months post-operatively.

Patients with class I AS showed greater pre-operative deviation (*p* = 0.01) than patients with other genotypes, while the type of strabismus (constant / intermittent, V-pattern, alternating) was no significantly different between AS classes (all *p* > 0.05).

Table 2 shows the gross and fine motor ability scores, assessed both before and after surgery. Assessment was only available for 14 patients pre-operatively and 10 patients post-operatively.

Overall, the median pre- and post-operative motor ability scores were: LyR 99 (IQR: 88–100) and 94 (IQR: 82–100), Si 90 (IQR: 71.5–100) and 85 (IQR: 73–99), CK 71 (IQR: 7–86) and 79 (IQR: 7–83.5), St 68 (IQR: 14.5–74.8) and 72 (IQR: 36–73), WRJ 35.5 (IQR: 11.8–53.8) and 50 (IQR: 25–56.5), G-TS 71.5 (IQR: 39.3–83.3) and 69 (IQR: 48–82), B-TS 27 (IQR: 24.8–38.5) and 30 (IQR: 25.3–38.5).

Subject 15 (female, 9 years old, class IV) showed the best gross and fine motor performance, while Subject 5 and Subject 9 (females, 9 years old, class I) showed the worst gross and fine motor performances, respectively.

No significant gender difference was found when examining motor ability scores (all *p* > 0.05).

Patients with class I AS showed worse pre-operative motor abilities than patients with other genotypes, but the difference was found to be statistically significant only for B-TS (*p* = 0.02).

Pre-operative motor abilities were found to improve with age in patients with class I AS. The relation was found to be statistically significant in Si (rs = 0.80, p < 0.01), CK (rs = 0.84, p < 0.01), St (rs = 0.76, p < 0.01), WRJ (rs = 0.61, p = 0.04), and G-TS (rs = 0.77, p < 0.01). Astigmatism was found to be negatively correlated to pre-operative motor abilities. The correlation was statistically significant in LyR (r_s_ = -0.61, *p* = 0.02), CK (r_s_ = -0.59, *p* = 0.02), St (r_s_ = -0.56, *p* = 0.03), and G-TS (r_s_ = -0.53, *p* = 0.05). The spherical correction and the spherical equivalent were found to be not significantly correlated to motor ability scores (all *p* > 0.05), while, anisometropia was found to be significantly negatively correlated to pre-operative G-TS (r_s_ = -0.54, *p* = 0.05). Patients with greater deviation showed worse pre-operative motor ability scores. The relation was statistically significant in only CK (r_s_ = -0.72, *p* < 0.01).

We found that all the pre-operative motor ability scores were significantly related with each other (all *p* < 0.05), except B-TS and CK.

Patients with class I AS showed also worse post-operative motor ability scores than patients with other genotypes, but the difference was found to be statistically significant only for post-operative Si (*p* = 0.03), and CK (*p* = 0.03).

Post-operative B-TS was found to be negatively correlated to astigmatism (r_s_ = -0.69, *p* = 0.03) and post-operative G-TS was found to be significantly negatively correlated to anisometropia (r_s_ = -0.70, *p* = 0.03). Patients with greater deviation showed also worse post-operative motor ability scores. The correlation was statistically significant in only CK (r_s_ = -0.78, *p* = 0.01).

Statistically significant differences in WRJ (*p* = 0.02) and B-TS (*p* = 0.03) were found between pre-operative and post-operative motor ability scores. No statistically significant difference in pre- and post-operative motor ability scores was found between patients who achieved and didn’t achieve orthotropia after surgery (all *p* > 0.05).

## Discussion

Angelman syndrome is a rare genetic neuro-behavioural disorder. While the clinical and neuro-behavioural profiles of AS are relatively well characterized, less is known about the ocular manifestations of AS and their possible treatment [12–16]. It’s been reported that AS patients frequently present with ametropia and strabismus [16], but little information is currently available about their correction [26].

In our cohort, the I, II, III and IV class of genetic mechanisms causing AS showed a prevalence of 70.6%, 5.9%, 5.9%, and 17.6%, respectively, with no gender differences. Our results are in line with the data in the literature, where class I has been reported to be the most common AS genotype, followed by class IV, III, and II [1, 7].

Out of 17 patients, 15 (88.2%) presented a refractive error greater than 1 D: 1 was hyperopic and 14 astigmatic, compound hyperopic astigmatism being the most frequently reported refractive error. Astigmatism wasn’t related with age, and was no significantly different between genetic classes; no case of keratoconus was observed. Our results are in accordance with the ones reported in previous studies [16, 26, 29]. Michieletto et al. [16] found that the prevalence of ametropia among 34 AS patients was 97%, astigmatism being the most commonly observed refractive error, regardless of age; Micheletti et al. [29] stated that all the 10 children in their study had ametropia, mainly hyperopic astigmatism; and all 3 cases reported by Ye et al. [26] presented with astigmatism.

The prevalence of nystagmus in our cohort was 5.9% (1 patient), slightly inferior to the one reported in the literature, which varies between 9% and 13% [12, 16, 36].

In our cohort, exotropia was the most common type of deviation, affecting 14 (82.4%) patients, and subjects with class I AS showed the greater pre-operative deviation angle. The type of strabismus, however, was not significantly different between AS classes. The high prevalence of strabismus, and especially exotropia, among AS patients with strabismus has already been reported by other authors [16, 29], whereas there are no data in the literature describing the relation between pre-operative deviation and AS genotype.

Even though the appropriate type and amount of surgery to correct strabismus has already been established in the currently available guidelines [32], some authors have suggested that the standard surgical tables may not be suitable to children with developmental delays [27]. In particular, a larger surgical effect per mm in children with developmental disorders has been reported, suggesting that, in these patients, under-correction is more likely to give better results [18–21]. However, in the scientific literature there is no consensus on the most suitable surgical dosage adjustment in these patients; and some authors noticed that reducing the amount of surgery in delayed children (mostly esotropic [22–25] but also exotropic [25]) to prevent overcorrection, is associated with a high rate of surgical failure, mainly due to undercorrection. Nevertheless, the same authors also observed that, on long term follow-up, some of these undercorrected esotropic patients drifted towards exotropia [22–24].

With regards to AS, only Ye et al. [26] reported a case series of 3 children undergoing correction of exotropia: bilateral LR recession (7.5 and 8 mm recession for 50 and 60 PD exotropic correction, respectively) under general anesthesia was performed, and the authors referred good postoperative outcomes.

The surgical outcomes in our cohort were variable according to the type and the amount of pre-operative strabismus. No case presented with exaggerated surgical response.

All 3 esotropic patients of our cohort achieved post-operative orthotropia.

The exotropic patients of our cohort achieved a variable post-operative reduction in the amount of pre-operative deviation, with poorer outcomes in subjects with constant strabismus and baseline deviation greater than 30 PD. Hence, according to our results at 1-year follow-up, we believe that the standard surgical tables should be applied to correct exotropia in AS patients, without reducing the amount of surgery. In particular, in accordance to our data and nomograms, 8 mm bilateral LR recession is indicated for 30–35 PD exotropic correction, while it is necessary to associate a unilateral MR resection for correction of 40–45 PD exotropic deviations. Indeed, we found consistency between baseline and post-operative deviation.

Even though the children investigated were unable to complete sensory fusion tests, binocular single vision was expected in all patients with intermittent strabismus. As fusional mechanisms may influence the surgical outcome, not surprisingly, all subjects of our cohort with intermittent exotropia achieved post-operative orthotropia. In this regard, given the amount of surgery performed and the good surgical outcome, it’s likely that all the 3 cases reported by Ye et al. [26] presented with intermittent exotropia.

Finally, the data of Subject 6, who showed a good surgical outcome at 3 months, which worsen at 6 months follow-up, is not surprising, since, Zehavi-Dorin et al. [22] already found that, in a number of cases, it is difficult to achieve a stable long-term ocular alignment in delayed children, though the authors analysed esotropic patients, while Subject 6 had exotropia.

Concerning anesthesiological issues, even tough, according to some authors [28], AS patients may be at higher risk of complications, our patients well tolerated the anaesthetic management, similarly to the subjects investigated by Ye et al. [26].

With regards to motor abilities, our patients showed significant delays in all GMFM and BSID-III domains, with baseline median G-TS and B-TS scores of, respectively, 71.5 (IQR: 39.3–83.3) and 27 (IQR: 24.8–38.5). Some authors already reported that AS children have a distinct developmental profile [30, 31]; with significant gross and fine motor delay [7, 37, 38], and less severe cognitive and language skills delay. Among the few studies currently available, Micheletti et al. [29] found a median G-TS of 80 (IQR: 66–84.3) in 10 AS children aged between 5 and 11 years; Gentile et al. [30] evaluated the BSID-III scores of 92 AS children aged between 5 months and 5 years, and reported a mean gross and fine motor skills developmental quotient of respectively 33.6 ± 10.4 and 32.5 ±13.6 in class I patients, 42.8 ± 15.1 and 42.1 ± 18.8 in class II-IV patients; and Peters et al. [31] found a mean B-TS of 48.76 ± 18.53 (range: <20–84) in 17 AS children younger than 6 years.

Children in our cohort presented a moderate impairment in crawling, kneeling and standing abilities and a more severe impairment in walking and fine motor tasks. Class I AS subjects showed the worse performance, but the difference was found to be statistically significant only for the fine motor tasks. Similarly to our study, Micheletti et al. [29] noticed that patients presented with a mild impairment in standing ability and a more severe impairment in walking tasks; with class I AS subjects showing the lowest scores. Also Gentile et al. [30] reported that class I children showed the lowest scores in both gross and fine motor development.

Regarding the age-related changes of AS patients’ developmental profile, we found that gross motor abilities (G-TS) appear to slightly improve with age only in patients with class I AS. Also, Beckung et al. [39] reported a little tendency towards more advanced motor function in AS patients at higher ages. In the case series, the improvement was more pronounced for fine motor function than for gross motor ability. This slight age-related improvement of motor abilities in subjects with AS doesn’t affect our results, as the post-operative gross and fine motor development assessment took place only 3 months after surgery.

Comparing ocular findings and motor abilities in our cohort, we found that gross motor abilities (G-TS) were negatively correlated to astigmatism, anisometropia, and deviation (the latter correlation being significant only for CK ability) at baseline. Conversely, no significant association was found between fine motor abilities (B-TS), refractive error and deviation.

Anamnestic data revealed that, immediately after the operation, most of our patients’ parents noticed a remarkable improvement in movement capabilities, especially going downstairs. These observations were confirmed by the data of the motor development assessment. Indeed, 3 months post-operatively, children presented a significant improvement in walking and also in fine motor tasks, thanks to reduction of the amount of deviation alone. In fact, no statistically significant difference was found between patients who achieved and didn’t achieve orthotropia after surgery. Moreover, post-operative gross motor abilities (G-TS) were still negatively correlated to deviation (the correlation being significant only for CK ability). These correlations, detected both pre- and post-operatively, proves that the amount of deviation angle is among the primary factors affecting the development of motor abilities. As already postulated by Drover et al. [17] for healthy children, and hypothesized by Micheletti et al. [29] for children with AS, our data suggest that strabismus surgery has a positive effect on gross and fine motor development in AS patients, with results depending to the amount of residual strabismus, better in patients who achieved a smaller post-operative deviation.

Post-operatively, gross motor abilities were still found to be negatively correlated to astigmatism and anisometropia, confirming that ametropia (notably astigmatism, which is the most common refractive error, with a prevalence of 93.3%) affects motor development in AS children and demonstrating that motor abilities improvement is mainly related to the reduction in the amount of baseline deviation angle. Unfortunately, often AS children don’t wear spectacles permanently, as prescribed. Our data confirm the importance of early diagnosis and prompt treatment of ocular alterations, as part of the rehabilitative program of AS patients [16].

## Conclusion

This study is the first to report and evaluate strabismus surgery in children with AS, in order to optimize and standardize surgical approach, formulating a predictable nomogram. Furthermore, this study represents the first attempt to understand the possible relation between ocular findings and motor ability, and between improvement in ocular alignment and changes in gross and fine motor skills in this population.

Unfortunately, the small sample size, related to the objective difficulties in collecting reliable data in this cohort of subjects with developmental delay, does not allow for definite conclusions. However, according to our data, the standard nomograms for strabismus surgery may be successfully applied to correct exotropia in AS patients. Moreover, since both deviation angle and astigmatism were found to negatively correlate with gross motor abilities in AS subjects, and motor functions, namely walking ability and fine motor tasks, significantly improved after surgery, our data suggest that early diagnosis and prompt treatment of ocular motility and refractive disorders positively influence motor development, and have, therefore, to be part of the rehabilitative program of AS patients.
